# Preoperative metabolic–inflammatory profiles and early postoperative recovery after immediate breast reconstruction in patients with and without neoadjuvant chemotherapy: a retrospective cohort study

**DOI:** 10.3389/fendo.2026.1921109

**Published:** 2026-10-26

**Authors:** Fan Wang, Lan Yang, Wei Bai, Chengyu Zhao, Yongmin Miao, Wenfu Yang, Yang Liu

**Affiliations:** 1 Department of Breast Surgery, Shanxi Cancer Hospital; Cancer Hospital of Chinese Academy of Medical Sciences (Shanxi Hospital); Affiliated Cancer Hospital of Shanxi Medical University, Taiyuan, Shanxi, China; 2 Department of Radiation Therapy, Thoracic Radiotherapy Ward III, Shanxi Provincial Cancer Hospital / Shanxi Hospital, Cancer Hospital, Chinese Academy of Medical Sciences / Third People's Hospital Affiliated to Shanxi Medical University, Taiyuan, Shanxi, China

**Keywords:** breast reconstruction, C-reactive protein, neoadjuvant chemotherapy, neutrophil-to-lymphocyte ratio, postoperative recovery, serum albumin

## Abstract

**Background:**

Neoadjuvant chemotherapy (NAC) may influence systemic metabolic-inflammatory status before surgery; however, the association between preoperative biomarker profiles and early recovery after immediate breast reconstruction remains incompletely understood.

**Methods:**

In our retrospective study, we included 214 patients who had immediate breast reconstruction; out of them, 102 patients received NAC, and the other 112 patients didn’t. Preoperative measurements of serum albumin, C-reactive protein (CRP), and neutrophil-to-lymphocyte ratio (NLR) were performed. The main outcome was prolonged hospitalization, defined as a length of stay (LOS) greater than the cohort median of 8 days; postoperative complications were evaluated separately. Factors associated with prolonged hospitalization were analyzed using multiple logistic regression, and the model’s discrimination was assessed using receiver operating characteristic (ROC) analysis.

**Results:**

Overall, 93 patients (43.5%) experienced prolonged hospitalization. Patients who received NAC were hospitalized longer than those who didn’t (8.6 ± 2.9 days vs 7.2 ± 2.4 days; P = 0.001), and NAC patients also had more complications (28.4% vs 17.0%; P = 0.045). In the multivariable analysis, NAC exposure (OR = 1.82, 95% CI: 1.08–3.05; P = 0.024) and a higher NLR (OR = 1.36, 95% CI: 1.12–1.65; P = 0.002) were independently related to longer length of stay, while a greater level of albumin was negatively related to length of stay (OR = 0.94, 95% CI: 0.90–0.98; P = 0.006). The multivariable model had fair discrimination (AUC = 0.742, 95% CI: 0.676–0.808).

**Conclusions:**

Preoperative albumin and NLR levels may help identify patients at greater risk of prolonged hospitalization; further study is needed for multicenter validation.

## 1 Introduction

Neoadjuvant chemotherapy (NAC) is a critical component of multidisciplinary care and provides a variety of clinical benefits in breast cancer management, including tumor downstaging, greater surgical resectability, and more options for breast-conserving surgery and immediate reconstruction. With the rise in the use of immediate breast reconstruction, perioperative optimization has become a key component of comprehensive breast cancer management. Even though NAC provides substantial oncological benefits, patients previously treated with NAC may exhibit systemic metabolic and inflammatory differences that can contribute to early recovery after surgery. Therefore, identifying readily identifiable biomarkers associated with perioperative outcomes may help enhance risk assessment and tailor perioperative management (1–4).

Chemotherapy may be associated with metabolic alterations, including increased protein catabolism, disrupted energy metabolism, and reduced physiological reserves, which may affect postoperative recovery in susceptible patients. These metabolic changes may impede healing, delay recovery, and prolong hospital stay for patients undergoing complex reconstructions. Serum albumin (ALB), which is commonly assessed in clinical settings, has long been assumed to be a marker pertaining to nutritional status and physiological reserve. However, albumin levels are also affected by systemic inflammation, hepatic production, hydration status, and illness severity. Hence, serum albumin must be considered an unspecific marker reflecting the state of general physiology rather than merely a quantitative indicator of nutritional status. Past studies have shown the correlation of low albumin levels with a higher incidence of postoperative complications and longer postoperative length of stay in different groups of patients undergoing surgical treatment for cancer. However, the impact of preoperative albumin levels on patients undergoing immediate breast reconstruction after NAC is still poorly studied (5–9). Additionally, systemic inflammation and metabolic changes create a synergistic biological system that affects perioperative recovery; surgical stress may activate inflammatory pathways via chemotherapy-induced tissue injury, leading to increased levels of acute-phase reactants and changes in circulating immune cell populations.

Systemic inflammation is another key factor relating to postoperative recovery. C-reactive protein (CRP) indicates the level of inflammatory activity, and the neutrophil-to-lymphocyte ratio (NLR) reflects the interplay between the innate inflammatory response and the regulatory immune response in adaptive immunity. Abnormalities of both biomarkers correlated with complications and processes of postoperative recovery in different types of surgery and cancer treatment. Nevertheless, it is also important to consider metabolic status and inflammation as intimately related processes that help explain how the patient responds physiologically to cancer treatment and surgery. The combination of these two markers enables the acquisition of more information about the patient’s physiological condition during the perioperative period, rather than acting as independent predictors of the outcome (10–17).

Patients undergoing immediate breast reconstruction post-mastectomy form a distinct cohort that requires good wound healing, an effective flow of blood to tissues surrounding the surgery site, appropriate inflammatory processing, and adequate physiological reserves. Unlike mastectomy done alone, immediate breast reconstruction takes longer in terms of surgical procedure, deals with more tissue, and adds more physiological strain that can increase susceptibility to post-surgery complications. Therefore, understanding which factors are important for a speedy recovery after breast reconstruction can help in identifying the need for additional monitoring for patients.

Although NAC has been intensely researched in terms of oncological results and responses, there is still not much knowledge related to how preoperative metabolic-inflammation factors influence postoperative recovery after immediate breast reconstruction. In particular, the importance of common laboratory indicators such as serum albumin, CRP, and NLR has to be considered.

This retrospective cohort research paper looked at whether there was a link between metabolic markers before surgery and the results obtained during the first few days after surgery, specifically in patients who had breast reconstruction with or without prior chemotherapy. Accordingly, the influence of NAC as well as the role of albumin, CRP, and NLR in determining the length of hospitalization and complications in the immediate postoperative period in patients who have undergone breast reconstruction was examined.

## 2 Materials and methods

### 2.1 Study design and setting

A retrospective observational cohort study was designed to assess the relationship of preoperative inflammatory-metabolic profiles to early postoperative recovery of patients having immediate breast reconstruction subsequent to mastectomy due to pathologically confirmed breast cancer. The study used routinely collected data, including demographic and clinical information, laboratory results, operative reports, and postoperative data, extracted from the institutional electronic medical record (EMR) system.

The study was undertaken at the Department of Breast Surgery, the Shanxi Cancer Hospital; the Cancer Hospital of the Chinese Academy of Medical Sciences (Shanxi Hospital); and the Affiliated Cancer Hospital of Shanxi Medical University, which is a very high-volume, tertiary referral center in Northern China that specializes in comprehensive breast cancer diagnosis, surgical treatment, and reconstruction.

The retrospective observational cohort design was selected because it allowed evaluation and analysis of real-world perioperative outcomes and experiences relative to existing clinical data, without influencing how any patient was managed, how any surgical decision was made, how any perioperative care was provided, or how any patient was followed after surgery. All patients received routine treatment throughout the study, and none underwent any diagnostic or therapeutic procedures directly related to the research project.

The study period ran from January 2018 to December 2023, during which sufficient clinical data were collected to examine preoperative metabolic-inflammatory biomarkers and the progression of early postoperative outcomes in patients undergoing immediate breast reconstruction. Predefined eligibility criteria were used to determine which patients would be included based on a review of clinical records, operative reports, laboratory evaluations, and hospitalization data, thereby ensuring that patients were identified consistently, data were extracted uniformly, and the analysis was replicable.

The study was conducted in accordance with the STROBE (Strengthening the Reporting of Observational Studies in Epidemiology) criteria to enhance transparency, completeness, and quality of reporting for such studies.

Anonymized patient records were used for all analyses, and all patient identifiers were removed prior to analysis to protect patient confidentiality and remain consistent with institutional data protections.

### 2.2 Study population

Between January 2020 and January 2026, patients who underwent immediate breast reconstruction following mastectomy were identified from the institutional database, including those who had previously received neoadjuvant chemotherapy (NAC) and those who had not. Out of the overall 214 eligible patients identified in this study (after applying exclusion/inclusion criteria), 102 were included in the neoadjuvant chemotherapy category, and 112 were included in the non-neoadjuvant chemotherapy category.

All eligible participants were adult female patients (≥ 18 years of age) who had been diagnosed with histologically confirmed breast cancer and underwent immediate breast reconstruction (as part of their treatment) at our facility, and had complete perioperative clinical and laboratory data available (for perioperative analysis). Patients were excluded if an active infection was documented in the preoperative medical record at the time of surgery, based on the treating clinical team’s diagnosis and/or documented clinical evidence of infection requiring antimicrobial treatment. Reconstruction techniques were classified as implant-based or autologous based on surgical records. Implant-based reconstruction is defined as surgical reconstruction performed with breast implants or tissue expanders, while autologous reconstruction is performed with the patient’s own tissue. Details and materials used in each reconstruction category were not sufficiently documented in most cases to conduct subgroup analyses. Moreover, additional exclusions were applied when crucial laboratory data were missing, or clinical outcome records were insufficient.

This study focused on early postoperative recovery, so the main observation period spanned from the date of surgery to the day of hospital discharge. Any postoperative complications noted during hospitalization or in the immediate postoperative period were included. Long-term clinical outcomes, such as recurrence, survival, and other long-term endpoints, are beyond the scope of this study.

### 2.3 Data collection

Clinical and demographic details were taken from the electronic health records. The variables included age, body mass index (BMI), tumor features, treatment history, neoadjuvant chemotherapy, surgical details, laboratory measurements, and post-surgical outcomes.

Laboratory inflammatory and metabolic markers were measured as part of the routine preoperative blood tests performed no later than 7 days before the surgery. For patients undergoing NAC, these laboratory findings were made after completion of NAC and before surgical intervention. For patients without NAC exposure, laboratory tests were taken during the routine preoperative evaluation before surgery. Laboratory findings reflected the immediate metabolic-inflammatory state before reconstructive surgery rather than changes during chemotherapy.

For patients given NAC, preoperative lab tests were performed after NAC and within 7 days before surgery. Unfortunately, the precise time frame between the last NAC cycle and preoperative blood sampling, or between the end of NAC and surgery, was not uniformly recorded in the retrospective records and was therefore omitted from the adjustment variables. The influence of differences in these time intervals on the study findings is recognized as one of the study’s limitations.

All gathered patient data have been anonymized prior to statistical analysis to ensure confidentiality and comply with institutional data protection regulations.

### 2.4 Reconstruction characteristics

The surgical records were examined in order to extract relevant reconstruction variables. The recorded details included the type of reconstruction (i.e., whether it was implant-based or autologous), the duration of the operation, the estimated blood loss, the side of the surgery, and reconstruction-related complications, such as flap necrosis, flap infection, and revision surgery, where applicable.

Reconstruction using implants meant carrying out the artificial reconstruction following a mastectomy, while the autologous reconstruction method meant reconstructing the breast using the tissue of the patient. Regarding the technical materials used, both methods differed based on clinical and surgical reasoning and were not documented correctly for further analysis.

Even when a number of variables related to reconstruction were available for both descriptive and between-group comparisons, some surgical and perioperative variables lacked sufficient completeness and granularity to be fully incorporated into the multivariable model. Consequently, the possibility of residual confounding due to the complexity of reconstructive surgery and perioperative measures taken cannot be excluded. It should also be noted that the possible effects of the degree of reconstruction, the time of operation, and other factors on the recovery process are mentioned in the Limits section.

### 2.5 Assessment of preoperative metabolic-inflammatory biomarkers

The systemic immune and inflammatory status of each patient was assessed using routinely measured laboratory-derived hematological variables from preoperative blood specimens. The biomarkers under examination were serum albumin, C-reactive protein (CRP), leukocyte count, absolute neutrophil count, and lymphocyte count.

NLR was calculated as the absolute neutrophil count divided by the absolute lymphocyte count.

CRP was assessed through the routine automated immunoturbidimetric method at the hospital laboratory, while serum albumin was evaluated through standard automated biochemical methods. All laboratory measurements adhered to the standard operating procedures of the institutional clinical laboratory and to reference ranges appropriate for the study duration.

Before statistical analysis, the continuous laboratory variables were checked for implausible or extreme results. Suspicious values that were likely to be data entry errors were validated against source medical records and amended if required. However, all other extreme observations were used in the later analyses. The reference ranges offered by the institution for the biomarkers studied in this research were: serum albumin 35.0-52.0 g/L; CRP<8.0 mg/L; white blood cell count 3.5-9.5 ×10^9^/L; absolute neutrophil count 1.8-6.3 ×10^9^/L; absolute lymphocyte count 1.1-3.2 ×10^9^/L. Laboratory values were treated as continuous variables rather than dichotomized based on the specified reference values.

CRP was treated as a measure of systemic inflammatory activity, while serum albumin was regarded as a non-specific marker reflecting the overall physiological condition of the body and was found to be perturbed by inflammation, hepatic synthesis, hydration, and disease severity. Preoperative CRP measurements were available for all 214 included patients (100%); therefore, there were no missing CRP data in the analytic cohort.

### 2.6 Outcome measures

The outcome of interest was prolonged hospitalization, defined *a priori* as an LOS exceeding the cohort median. The cohort median LOS was 8 days. Accordingly, LOS that was greater than 8 days was classified as prolonged hospitalization. LOS was chosen as a measure of early post-operative recovery following immediate breast reconstruction.

The secondary outcomes included early post-operative complications such as wound infection, delayed wound healing, and reoperation in relation to breast reconstruction. The purpose of this analysis is to avoid combining different clinically significant postoperative events into a single endpoint.

### 2.7 Statistical analysis

Statistical analysis was performed using SPSS version 22.0 (IBM Corp., Armonk, NY, USA). Continuous variables are presented as mean ± standard deviation (SD) or median (interquartile range [IQR]), as appropriate, while categorical variables are presented as frequencies and percentages. Comparisons between the neoadjuvant chemotherapy (NAC) and non-NAC groups were performed using the independent-samples t-test or Mann–Whitney U test for continuous variables and the chi-squared test or Fisher’s exact test for categorical variables, as appropriate.

Because of baseline differences between groups, propensity score matching was considered; however, formal matching was not feasible because several potentially important covariates were not consistently available in this retrospective dataset. To minimize potential confounding variables, multiple-variable analyses were conducted using clinically important variables from the dataset.

Before the analysis, the amount of missing data for each variable was assessed. An analysis using only complete cases was used when there is less than 5% of missing values in a given variable.

A univariate logistic regression model was used to find the relationship between the candidate variables and longer-than-average hospitalization, which was defined as longer than 8 days.

Multivariable analysis variables were selected not only on statistical grounds but also on analytical grounds.

Finally, multivariable logistic regression was applied to identify variables independently associated with longer-than-average hospitalization.

To verify model flexibility, the ROC curve and the area under the curve were used. The Hosmer–Lemeshow test was used to verify model precision.

The linearity-in-the-logit condition was checked with the Box–Tidwell test. BMI, NLR, serum albumin, and CRP met the linearity condition. However, age did not since the P-value was 0.027 when compared to the interaction variable. The analysis of different functional forms of age showed that none of them significantly improved model flexibility.

CRP met the linearity-in-the-logit assumption but was not included in the final model. Although the data show that CRP and NLR measure similar aspects of inflammatory activity, including them in a single model would complicate the overall model structure.

A two-tailed P-value< 0.05 was considered statistically significant.

### 2.8 Ethical approval

This study was conducted in accordance with the principles of the Declaration of Helsinki. Ethical approval was obtained from the Institutional Review Board of Shanxi Cancer Hospital, Cancer Hospital of the Chinese Academy of Medical Sciences (Shanxi Hospital), and the Affiliated Cancer Hospital of Shanxi Medical University (Approval No. YXLL-2023-010).

Given the retrospective and non-interventional design of the study, the requirement for written informed consent was waived by the Institutional Review Board. All patient data were anonymized and de-identified prior to analysis to ensure confidentiality.

## 3 Results

### 3.1 Baseline characteristics

Out of 250 patients screened for inclusion, 214 were eligible based on the predetermined exclusion/inclusion criteria established prior to study initiation. Of those included in the study, 102 received neoadjuvant chemotherapy (NAC), and 112 did not (non-NAC). Patients were removed from consideration if they had an active infection, an autoimmune disease, received chronic immunosuppressive therapy, did not have complete laboratory data, and/or did not have follow-up visit documentation. Patient selection criteria are shown in **Figure 1**.

**Figure 1:**
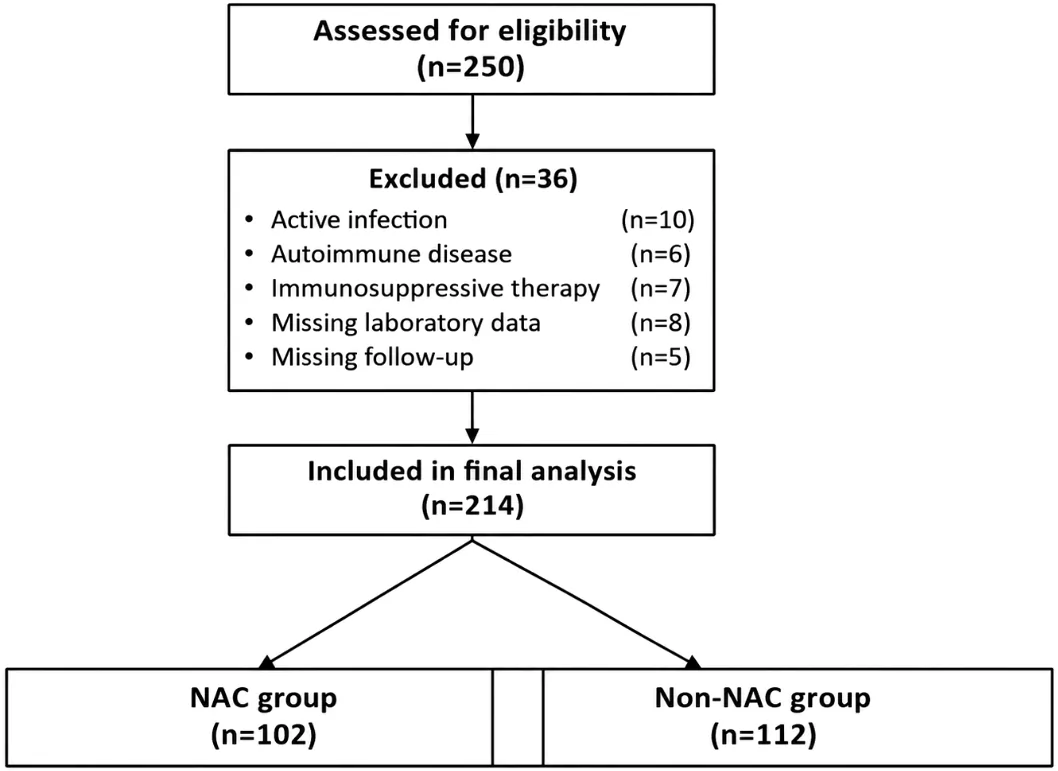
Study cohort flow diagram and patient selection process. This diagram details the components of patient screening, the eligibility evaluation, the exclusion criteria used, and how study participants were ultimately allocated to the study cohort for inclusion. A total of 214 patients met all eligibility criteria; 102 in the Neoadjuvant Chemotherapy (NAC) Group and 112 in the Non-NAC Group were eligible and enrolled in the study.

**Table 1** describes the pre-treatment clinicopathological characteristics of both patient groups. The two groups of patients were similar in clinical and demographic characteristics. There were no statistical differences between the groups with regard to age (49.8 ± 10.6 vs 51.2 ± 9.8 years; P = 0.278), BMI (23.6 ± 3.2 vs 23.9 ± 3.5 kg/m²; P = 0.512), % ER+ (62.7% vs 63.4%; P = 0.915), % HER2+ (37.3% vs 34.8%; P = 0.702) or % smokers (8.8% vs 9.8%; P = 0.801). However, significantly more patients in the NAC group presented with advanced-stage disease than in the non-NAC group (76.5% vs 61.6%; P = 0.018).

**Table 1:** Baseline characteristics.

Variable	NAC (n=102)	Non-NAC(n=112)	P-value
Age (years)	49.8 ± 10.6	51.2 ± 9.8	0.278
BMI (kg/m^2^)	23.6 ± 3.2	23.9 ± 3.5	0.512
Tumor Stage (II/III), n (%)	78(76.5%)	69(61.6%)	0.018*
Estrogen Receptor Positive, n%	64(62.7%)	71(63.4%)	0.915
HER2 positive, n (%)	38(37.3%)	39(34.8%)	0.702
Smoking Status (yes), n%	9(8.8%)	11(9.8%)	0.801

Data presentation consisted of mean ± standard deviation (SD) or numbers (percentages) of cases, where applicable. NAC means neoadjuvant chemotherapy; BMI means body mass index; HER2 means human epidermal growth factor receptor 2; P< 0.05 shows statistically significant results.

*P < 0.05 indicates statistical significance.

### 3.2 Surgical and reconstruction characteristics according to NAC exposure

Given that reconstruction-related factors may influence postoperative recovery and may differ by NAC exposure, we compared surgical and reconstruction characteristics between the NAC and non-NAC groups (**Table 2**). There were no substantial discrepancies in reconstruction type, surgical laterality, operative duration, estimated blood loss, or complications associated with the reconstruction process between the two groups.

**Table 2:** Surgical and reconstruction characteristics according to neoadjuvant chemotherapy exposure.

Variable	NAC (n=102)	Non-NAC (n=112)	P-value
Reconstruction type, n (%)			
Implant-based reconstruction	58 (56.9%)	70 (62.5%)	0.414
Autologous reconstruction	44 (43.1%)	42 (37.5%)	0.414
Surgical Laterality, n (%)			
Unilateral reconstruction	86 (84.3%)	96 (85.7%)	0.778
Bilateral reconstruction	16 (15.7%)	16 (14.3%)	0.778
Opérative duration (minutes)	286.4 ± 58.7	278.9 ± 55.3	0.355
Estimated blood loss (mL)	186.5 ± 72.4	178.2 ± 68.9	0.404
Flap-related complications, n (%)			
Flap necrosis	6 (5.9%)	5 (4.5%)	0.661
Flap infection	7 (6.9%)	5 (4.5%)	0.465
Revision surgery related to reconstruction	5 (4.9%)	4 (3.6%)	0.670

Data are presented as mean ± standard deviation (SD) or number (percentage), as appropriate. NAC, neoadjuvant chemotherapy. P< 0.05 was considered statistically significant.

Implant-based reconstruction was used in 56.9% of patients in the NAC group and 62.5% in the non-NAC group (P = 0.414), and autologous reconstruction was used in 43.1% of patients in the NAC group and 37.5% in the non-NAC group (P = 0.414). As for unilateral reconstruction, there were no substantial differences between the groups (84.3% vs 85.7%; P = 0.778), as well as in bilateral cases (15.7% vs 14.3%; P = 0.778).

Operative duration (286.4 ± 58.7 vs 278.9 ± 55.3 minutes; P = 0.355) and estimated blood loss (186.5 ± 72.4 vs 178.2 ± 68.9 mL; P = 0.404) were also similar between the groups examined. In addition, there was no notable difference between the groups in flap necrosis, flap infection, or revision surgeries due to the influence of reconstruction.

### 3.3 Preoperative metabolic-inflammatory biomarker profiles

NAC exposure-related preoperative metabolic-inflammatory marker patterns are displayed in **Table 3** and **Figure 2**. Patients who received NAC exhibited a distinct preoperative metabolic-inflammatory profile compared with those who did not.

**Table 3:** Preoperative metabolic-inflammatory biomarker profile according to neoadjuvant chemotherapy status.

Biomarker	Physiological domain	NAC (n=102)	Non-NAC (n=112)	P-value
Metabolic reserve				
Serum albumin (g/L)	Nutritional and metabolic reserve	38.2 ± 4.1	40.1 ± 3.8	0.003
Inflammatory burden				
C-reactive protein (CRP) (mg/L)	Acute-phase inflammatory response	5.8 ± 2.4	4.6 ± 2.1	0.009
Neutrophil-to-lymphocyte Ratio (NLR)	Systemic Inflammatory Balance	2.60 ± 1.10	2.20 ± 0.90	0.004
White blood cell count (× 10/*L*)	Peripheral inflammatory cell count	6.1 ± 1.8	6.8 ± 1.7	0.006
Absolute neutrophil count (× 10/*L*)	Innate immune response	3.9 ± 1.4	4.5 ± 1.3	0.002
Absolute Lymphocyte Count	Adaptive immune response	1.5 ± 0.5	1.8 ± 0.6	0.001

Results are represented in mean ± SD format. To compare continuous variables, we used the independent-samples t-test or the Mann-Whitney U test. CRP stands for C-reactive protein, NLR stands for neutrophil-to-lymphocyte ratio, and WBC stands for white blood cell count. The p-value was considered statistically significant at p< 0.05.

**Figure 2:**
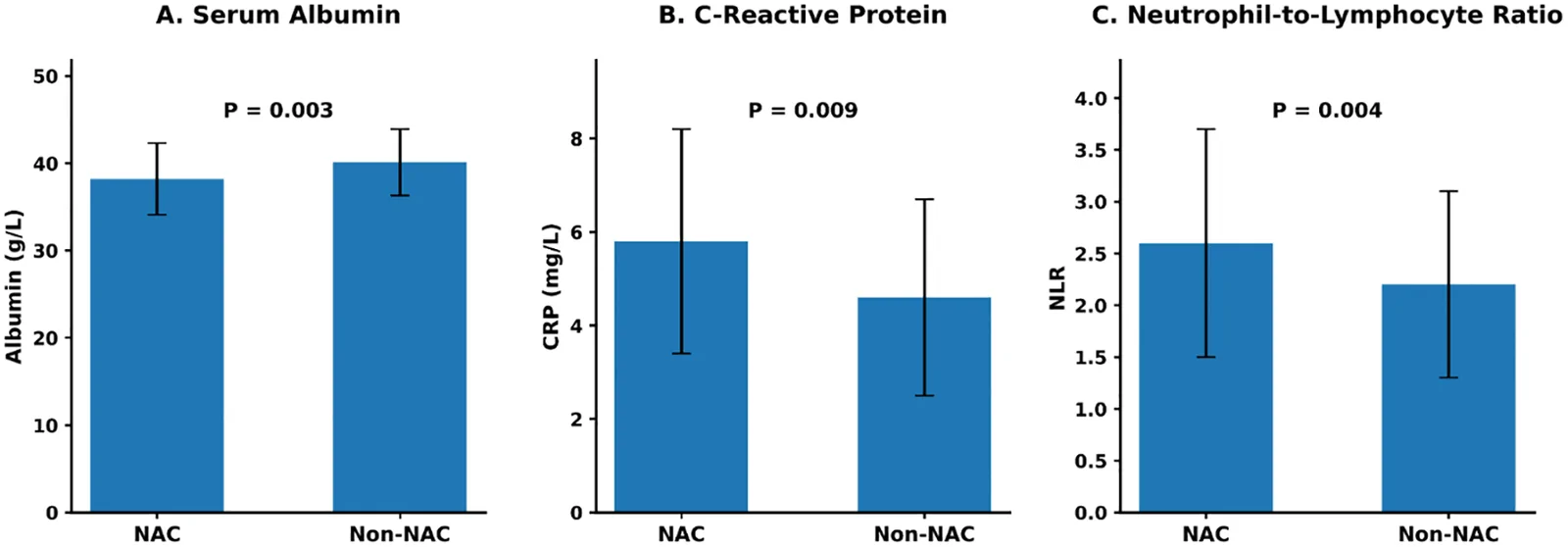
Comparison of perioperative metabolic-inflammatory biomarker profiles based on NAC status. Comparison of perioperative metabolic–inflammatory biomarkers for patients who received neoadjuvant chemotherapy (NAC) as compared with patients who did not. **(A)** Serum albumin, **(B)** C-reactive protein (CRP), and **(C)** Neutrophil-to-lymphocyte Ratio (NLR). Boxplots depict the median, interquartile range, and overall distribution of each metabolic–inflammatory biomarker. Statistical comparisons utilized an independent-samples t-test.

NAC users displayed significantly lower serum albumin concentrations than individuals who did not use NAC (38.2 ± 4.1 vs 40.1 ± 3.8 g/L; P = 0.003). Patients receiving NAC had higher CRP values than those not receiving NAC (5.8 ± 2.4 vs 4.6 ± 2.1 mg/L; P = 0.009). NLR was also significantly higher among patients receiving NAC than among those not receiving NAC (2.60 ± 1.10 vs 2.20 ± 0.90; P = 0.004).

NAC users also had significantly lower WBC, ANC, and ALC values than non-NAC patients.

In conclusion, NAC users had lower preoperative serum albumin levels and higher CRP and NLR values than non-NAC patients.

### 3.4 Early postoperative recovery outcomes

The outcomes of early postoperative recovery were assessed specifically for hospital length of stay and postoperative complications. The duration-of-stay outcomes are illustrated in **Table 4A** and **Supplementary Figure 1**, while postoperative complications are described in **Table 4B**.

**Table 4A:** Length of hospital stay and prolonged hospitalization according to neoadjuvant chemotherapy exposure.

Outcome	Overall (n=214)	NAC (n=102)	Non-NAC (N = 112)	P-value
Length of stay (days)	7.9 ± 2.7	8.6 ± 2.9	7.2 ± 2.4	0.001^*^
Cohort median LOS, days	8	–	–	–
Prolonged hospitalization (LOS >8 days), n (%)	93 (43.5%)	53 (52.0%)	40 (35.7%)	0.016*

Data are presented as mean ± standard deviation (SD) or number (percentage), as appropriate. Prolonged hospitalization was defined as LOS exceeding the overall cohort median of 8 days. NAC, neoadjuvant chemotherapy; LOS, length of stay. P< 0.05 was considered statistically significant. *P < 0.05 indicates statistical significance.

**Table 4B:** Postoperative complications according to neoadjuvant chemotherapy exposure.

Outcome	NAC (n=102)	Non-NAC (n=112)	P-value
Any Postoperative complication	29 (28.4%)	19 (17.0%)	0.045^*^
Wound Infection	14 (13.7%)	8 (7.1%)	0.098
Delayed wound healing	18 (17.6%)	9 (8.0%)	0.028^*^
Reoperation related to reconstruction	6 (5.9%)	4 (3.6%)	0.523

Data are presented as number (percentage). NAC, neoadjuvant chemotherapy. *P* < 0.05 was considered statistically significant. *P < 0.05 indicates statistical significance.

#### 3.4.1 Length of hospital stay

The median length of stay (LOS) for the whole cohort was 8 days. For this study, prolonged hospitalization was defined as length of stay (LOS) exceeding the median. A total of 93 out of 214 subjects (43.5%) met this definition for prolonged hospitalization. The number of patients seen in the NAC group with prolonged hospitalization was significantly higher than in the non-NAC group [53 of 102 treated patients (52.0%) vs 40 of 112 untreated patients (35.7%); P = 0.016]. In addition, the sample of patients receiving NAC had a significantly longer average LOS compared to patients not receiving NAC (8.6 ± 2.9 vs 7.2 ± 2.4 days; P = 0.001) (**Table 4A**; **Supplementary Figure 1**).

#### 3.4.2 Postoperative complications

The incidence of postoperative complications was higher among the NAC group compared to the non-NAC group (28.4% vs. 17.0%; P = 0.045) (**Table 4B**). Moreover, there was a significant difference in the rate of delayed wound healing between those who received NAC and those who did not (17.6% vs. 8.0%; P = 0.028). While there were numerical differences in the rates of wound infection and reoperation in the NAC group, their significance was not established.

In general, patients exposed to NAC prior to surgery had a significantly longer LOS as opposed to their counterparts not subjected to NAC.

**Figure 3:**
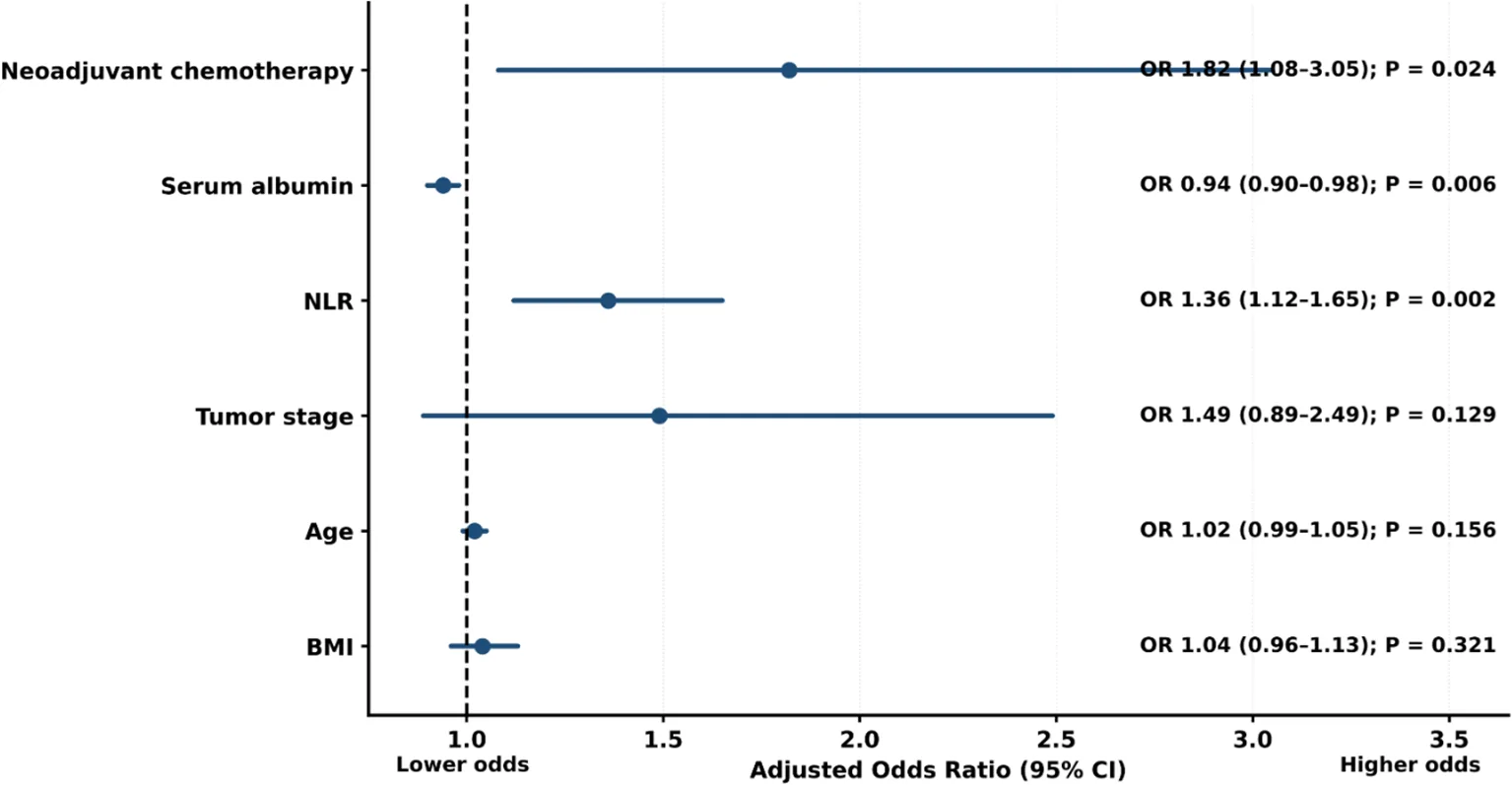
Multivariable logistic regression of factors associated with extended postoperative recovery following immediate breast reconstruction. Statistical dependent variable of adjusted odds ratios (ORs) and their associated 95% confidence intervals (CIs) of factors associated with an extended postoperative recovery period following immediate breast reconstruction. The vertical reference line indicates the OR = 1; ORs greater than 1.0 indicate an increased likelihood of prolonged recovery, while ORs less than 1.0 indicate a decreased likelihood of prolonged recovery.

### 3.5 Univariable and multivariable evaluation of prolonged hospitalization

In the unadjusted analysis, NAC exposure, higher NLR, higher CRP, advanced tumor stage, and lower serum albumin levels were significantly associated with prolonged hospitalization (**Table 5**). Age and BMI were additionally included in the multivariable model as clinically relevant covariates.

**Table 5:** Univariable logistic regression analysis of factors associated with prolonged hospitalization.

Variable	OR	95% CI	P-value
Neoadjuvant chemotherapy	1.94	1.13-3.33	0.016*
NLR (per unit increase)	1.42	1.18-1.71	<0.001^*^
CRP (per 1 mg/L increase)	1.24	1.08–1.42	0.002*
Age (per year)	1.03	1.00-1.06	0.081
BMI (kg/m^2^)	1.05	0.98-1.13	0.184
Tumor stage (III vs II)	1.67	1.02-2.74	0.041^*^
Albumin (per g/L)	0.93	0.89-0.97	0.002^*^

OR, odds ratio; CI, confidence interval; BMI, body mass index; NLR, neutrophil-to-lymphocyte ratio; CRP, C-reactive protein. Prolonged hospitalization was defined as a length of stay greater than the cohort median of 8 days. A univariable logistic regression was performed to identify factors associated with prolonged hospitalization. P< 0.05 is considered statistically significant. Bold values indicate statistical significant. *P < 0.05 indicates statistical significance.

The multivariable analysis results are represented in **Supplementary Table 1** and **Figure 3**. After controlling for the confounding variables in the model, NAC exposure continued to show a significant relationship with prolonged hospitalization (OR = 1.82; 95% CI: 1.08-3.05; P = 0.024).

Elevated NLR was linked with increased odds of protracted hospitalization (OR = 1.36; 95% CI: 1.12–1.65; P = 0.002), while higher serum albumin concentration was associated with decreased odds of protracted hospitalization (OR = 0.94; 95% CI: 0.90–0.98; P = 0.006).

Age (OR = 1.02; 95% CI: 0.99–1.05; P = 0.156), BMI (OR = 1.04; 95% CI: 0.96–1.13; P = 0.321), and tumor stage (OR = 1.49; 95% CI: 0.89–2.49; P = 0.129) were not statistically significant relevant to protracted hospitalization after controlling for other variables present in the model.

### 3.6 CRP selection in the multivariable model

In the univariable analysis, higher CRP levels were significantly associated with prolonged hospitalization (OR = 1.24 per 1 mg/L increase; 95% CI: 1.08–1.42; P = 0.002). In the final multivariable model, however, CRP was not retained. Since both CRP and NLR signal different aspects of systemic inflammatory activity, including multiple biomarkers with similar functions may make the models overly complicated and lead to overparameterization. Thus, NLR was chosen as the primary inflammatory biomarker for further analyses in the final multivariable model, based on the biological basis underlying its association with prolonged hospitalization.

### 3.7 Predictive performance of the multivariable model

The final multivariable model had moderate ability to discriminate, with an AUC of 0.742 (95% CI: 0.676–0.808), a sensitivity of 71.4%, and a specificity of 68.9%. The Hosmer–Lemeshow test for goodness-of-fit showed no indications of lack of calibration (χ² = 6.82; P = 0.556) (**Table 6**).

**Table 6:** Predictive performance of the multivariable logistic regression model for prolonged recovery.

Performance measure	Value
Area under ROC curve (AUC)	0.742
95% Confidence Interval	0.676-0.808
Sensitivity	71.4%
Specificity	68.9%
Hosmer-Lemeshow test _χ_2	6.82
Calibration P-value	0.556

AUC, area under the receiver operating characteristic (ROC) curve; CI, confidence interval. Model discrimination was assessed using ROC curve analysis and the AUC. Model calibration was assessed using the Hosmer–Lemeshow goodness-of-fit test.

## 4 Discussion

### 4.1 Principal findings and contextualization

The study identified differences in metabolic and inflammatory markers before surgery, as well as in early postoperative outcomes, between patients who received the helper chemotherapy and those who did not. There were lower levels of albumin among the patients who went through chemotherapy before undergoing surgery (38.2 ± 4.1 g/L vs 40.1 ± 3.8 g/L; P = 0.003), higher levels of CRP (5.8 ± 2.4 mg/L vs 4.6 ± 2.1 mg/L; P = 0.009), and increased NLR (2.60 ± 1.10 vs 2.20 ± 0.90; P = 0.004). Patients belonging to this group had longer hospital stays and experienced a higher number of postoperative complications. However, because this is a retrospective study, these results do not establish NAC’s causal role in mediating differences in biomarkers and recovery.

Multivariable analysis revealed that NAC exposure, together with increased NLR, was an independent predictor of a higher probability of prolonged hospitalization, whereas higher serum albumin levels were independently associated with a reduced probability of prolonged hospitalization. These results suggest that information on preoperative inflammation and physiological conditions may be useful for evaluating early postoperative risk.

Unlike previous studies that assessed individual inflammatory biomarkers or surgical outcomes, this study measured serum albumin, CRP, and NLR within a broader metabolic-inflammatory context; however, only albumin and NLR values were included in the final multivariable model. Thus, the results justify the use of these markers as adjunctive indicators of preoperative condition rather than as a proven jointly predictive model.

### 4.2 Metabolic–inflammatory alterations following neoadjuvant chemotherapy

Patients who receive neoadjuvant chemotherapy can experience complex physiological changes besides the response to the tumor. These changes may involve alterations in metabolic reserve, inflammatory activity, and host physiological adaptation. In this study, patients who received NAC had lower serum albumin levels and higher CRP and NLR values than those who did not receive NAC. The findings suggest differences in metabolic-inflammatory status before surgery among the groups, but they should not be construed as evidence that NAC caused these biomarker differences.

Serum albumin can indicate metabolic reserve and systemic physiological status, while CRP and NLR provide additional information on inflammatory load and inflammatory equilibrium, respectively (14–17).

Patients receiving NAC had lower serum albumin levels and higher CRP and NLR values than those who did not receive NAC. Higher serum albumin continued to be independently associated with a lower likelihood of prolonged hospitalization, while higher NLR remained an independent predictor of prolonged hospitalization after adjusting for relevant covariates (17–20).

Increased inflammatory activity combined with impaired metabolic reserve may be the reason for delay in recovery after immediate breast reconstruction. Thus, a combined evaluation of metabolic reserve and inflammatory activity can provide more clinical insights than looking at individual inflammatory biomarkers alone.

### 4.3 Albumin as a marker of metabolic reserve

Serum albumin is often regarded as a major indicator of changes in nutritional status, liver function, and systemic inflammation. In perioperative oncology, albumin is widely recognized as a significant biomarker reflecting a patient’s physiological state.

Serum albumin should not be viewed as a purely nutritional marker. Its interdependence with recovery likely results from a mix of inflammatory burden, physiological reserve, overall disease state, and an individual’s capacity to cope with surgical stress.

It seems that during systemic inflammation, the rate of albumin synthesis in the liver decreases while blood vessel permeability increases, leading to a decline in circulating albumin levels.

Based on the results of the study described in the current paper, patients treated with NAC had lower serum albumin levels than those receiving no NAC (14–20). The most striking aspect of this observation is that higher serum albumin was independently associated with a lower likelihood of prolonged hospitalization after adjustment for other factors.

The research results support the idea that albumin is a more significant indicator of host resilience than of mere nutrition. Thus, the piece of evidence mentioned above indicates that one should keep in mind the connection between albumin and inflammation when interpreting albumin in the perioperative context.

### 4.4 Clinical implications for perioperative management

The observed outcomes could have ramifications for the assessment of perioperative risks among individuals undergoing immediate breast reconstruction after NAC. The conclusion does not imply a causal relationship between the metabolic-inflammatory differences, but it indicates that such distinctions can reveal which patients are more likely to experience delayed postoperative recovery.

In a routine preoperative clinical setting, standard assays of readily available clinical markers, such as serum albumin and NLR, could provide more insights into identifying patients who require close attention and monitoring in the perioperative period. Not yet qualified as definitive predictive indicators, the biomarkers in question need to be validated via future research before being used in the practical health care system.

In our research, serum albumin showed an isolated protective association with postoperative recovery, whereas the elevated NLR maintained an independent association with delayed recovery following the correction. In this study, despite a noticeable difference in mean CRP levels between the NAC and non-NAC groups and a positive correlation of higher mean CRP with prolonged hospitalization in univariable analysis, the authors did not include CRP in the final multivariable model. Thus, this study could not affirm whether CRP has an independent association with prolonged hospitalization, after controlling for covariates that were included in the final model.

Patients with poorer preoperative metabolic and inflammatory profiles require careful perioperative monitoring; at the same time, prospective studies are needed to assess whether biomarker-based interventions, such as nutritional therapy, ERAS methodology, early mobilization, or improved monitoring, would improve patient outcomes. However, there is still no evidence that the discussed approaches improve clinical outcomes.

Therefore, it is crucial to conduct future prospective studies, which will help evaluate the effects of including metabolic-inflammatory biomarkers in the implementation of perioperative management protocols on recovery and complications after immediate breast reconstruction after NAC (21–23).

### 4.5 Comparison with existing literature

Prior research studying neoadjuvant chemotherapy for breast cancer has mostly examined either pathological response rates, oncologic outcomes, or post-operative complications, while few have been conducted investigating the perioperative metabolic and inflammatory changes, which may be linked to early recovery relative to immediate breast reconstruction (24–27).

Our results confirm previous reports showing that an elevated NLR is linked to poorer post-operative outcomes and reflects an overall systemic inflammatory state in populations of breast cancer patients and other cancer patients (28–30). Likewise, elevated CRP has also been found to be linked to increased post-operative morbidity and longer hospital stays after major surgery (30–32).

CRP levels were significantly different between the NAC and non-NAC groups, but while there was an association with prolonged hospitalization in the univariable analysis, it did not feature in the final multivariable analysis and, therefore, could not be said to demonstrate an independent relationship following multivariable correction. Rather, CRP can serve as additional information when combined with other indicators, particularly NLR and albumin.

The current findings suggest that lower serum albumin and greater systemic inflammatory activity form part of a preoperative profile associated with prolonged hospitalization.

### 4.6 Strengths and limitations

This study has several strengths. It is based on a well-documented cohort of patients receiving immediate breast reconstruction from an extremely busy tertiary cancer center, and looked at routinely measured metabolic/inflammatory biomarkers with direct clinical relevance. Furthermore, using multivariable logistic regression, the study evaluated factors independently associated with prolonged hospitalization while accounting for selected clinical covariates. The study also evaluated routinely available preoperative biomarkers within a metabolic-inflammatory framework, while recognizing that CRP was not retained in the final multivariable model.

Nonetheless, several limitations should be noted. First, the retrospective design of the study limits causal inference and increases the risk of selection bias and residual confounding.

Second, it was impossible to obtain pre-NAC biomarker measurements, which prevented evaluation of within-patient variations in metabolic-inflammatory status attributable to NAC. Thus, one cannot say that the differences in biomarkers were directly due to NAC.

In addition, although CRP was significantly associated with prolonged hospitalization in univariable analysis, it was not included in the final multivariable model; therefore, its independent association with prolonged hospitalization after adjustment for other covariates could not be determined.

Thirdly, even though multiple variables related to the reconstruction surgery, including the type of surgery, the time spent in surgery, estimated blood loss, and the side of surgery, were collected, several important surgical and peri-operative indicators were either omitted from the analysis or incompletely accounted for, which leaves some space for residual confounding.

Fourth, propensity score methods were considered but not applied because several potentially important confounders were measured only incompletely. Applying propensity score methods without adequate measurement of important confounders can lead to incomplete adjustment and potentially misleading estimates of group comparability. Fifthly, the exact intervals between NAC completion, preoperative blood sampling, and surgery were not consistently available. Consequently, potential variation in recovery time following chemotherapy and its influence on preoperative biomarker levels could not be formally evaluated.

Ultimately, the absence of serial biomarker readings and clinical threshold values hindered the evaluation of time-dependent biomarker changes and the determination of clinically applicable cut-off values.

Additional research involving multiple centers is essential to verify these results and assess the clinical usefulness of metabolic-inflammatory biomarkers in forecasting the recovery process after immediate breast reconstructive surgery.

### 4.7 Future directions and closing perspective

Future studies conducted in multiple centers are needed in order to determine the role of metabolic-inflammatory biomarkers in risk stratification of patients receiving immediate breast reconstruction post NAC.

In particular, further studies should assess clinically relevant cutoff values for serum albumin, CRP, and NLR, and investigate whether perioperative optimization interventions based on biomarker use (nutritional approaches and ERAS pathways) could improve recovery and minimize complications (33–35).

Although serum albumin, CRP, and NLR may provide relevant information for perioperative risk evaluation, their clinical applicability remains to be confirmed by predictive performance and further external validation in forthcoming studies (36–38).

## 5 Conclusion

Patients who underwent preoperative NAC before immediate breast reconstruction had a different metabolic-inflammation profile at baseline than patients without NAC. They had lower albumin levels and higher CRP and NLR, which in turn prolonged their length of hospitalization and increased postoperative complication rates. As the study was conducted retrospectively, associations presented should be interpreted as such rather than as proof of causation.

In the final multivariable analysis, the finding that NAC and high preoperative NLR were independent predictors of prolonged hospitalization is balanced by the finding that higher albumin was also an independent predictor of lower probability of prolonged hospitalization. CRP differed significantly between the NAC and non-NAC groups but was not retained in the final multivariable model, despite a significant association with prolonged hospitalization in the univariable analysis.

Prospective multicenter studies are required to validate these findings, determine clinically relevant biomarker thresholds, and demonstrate whether using these biomarkers in perioperative risk assessment improves outcomes.

## Data Availability

The original contributions presented in the study are included in the article/**Supplementary Material**. Further inquiries can be directed to the corresponding authors.
