# Supplementary material for: Preoperative metabolic–inflammatory profiles and early postoperative recovery after immediate breast reconstruction in patients with and without neoadjuvant chemotherapy: a retrospective cohort study
Source: Front Endocrinol (Lausanne). 2026 Oct 26;17:1921109. doi: 10.3389/fendo.2026.1921109 (PMC13745238; doi:10.3389/fendo.2026.1921109)
Supplement: Supplementary file 2 [file Table2.docx]

**Supplementary Table 1. Multivariable Logistic Regression Analysis of Factors Associated with Prolonged Hospitalization.**

| **Variable** | **OR** | **95% CI** | **P-value** |
| --- | --- | --- | --- |
| Neoadjuvant chemotherapy | 1.82 | 1.08-3.05 | 0.024* |
| NLR (per unit increase) | 1.36 | 1.12-1.65 | 0.002^*^ |
| Age (per year) | 1.02 | 0.99-1.05 | 0.156 |
| BMI (kg/m^2^) | 1.04 | 0.96-1.13 | 0.321 |
| Tumor stage (III vs II) | 1.49 | 0.89-2.49 | 0.129 |
| Albumin (per g/l) | 0.94 | 0.90-0.98 | 0.006^*^ |

OR denotes the odds ratio, CI the confidence interval, BMI the body mass index, and NLR the neutrophil-to-lymphocyte ratio. Prolonged hospitalization was defined as a stay longer than the overall median stay of 8 days for the entire group. The independent variables used in the multivariable logistic regression were: age, BMI, cancer stage, neoadjuvant chemotherapy, NLR, and serum albumin levels. A p-value of less than 0.05 was taken to represent statistical significance.
